# Chronic Spontaneous Urticaria Is Characterized by Lower Serum Advanced Glycation End-Products

**DOI:** 10.1155/2014/974154

**Published:** 2014-08-11

**Authors:** Alicja Grzanka, Aleksandra Damasiewicz-Bodzek, Edyta Machura, Magdalena Szumska, Krystyna Tyrpień-Golder, Bogdan Mazur, Alicja Kasperska-Zajac

**Affiliations:** ^1^Chair and Clinical Department of Internal Diseases, Dermatology and Allergology, Ulica M. Curie-Skłodowskiej 10, Medical University of Silesia in Katowice, 41-800 Zabrze, Poland; ^2^Department of Chemistry, Ulica Jordana 19, Medical University of Silesia in Katowice, 41-808 Zabrze, Poland; ^3^Chair and Department of Paediatrics, Ulica 3 Maja 13-15, Medical University of Silesia in Katowice, 41-800 Zabrze, Poland; ^4^Department of Microbiology and Immunology, Ulica Jordana 19, Medical University of Silesia in Katowice, 41-808 Zabrze, Poland

## Abstract

*Background.* Chronic spontaneous urticaria (CSU) is associated with activation of acute phase response. On the other hand, it is known that systemic inflammation may lead to increased formation of advanced glycation end-products (AGEs), associated with pathogenesis of various diseases. *Aim.* We aim to test whether chronic inflammation manifested by activated acute phase response may provide a mechanism for increased serum AGEs concentration in CSU. *Methods.* Concentrations of AGEs were measured spectrofluorimetrically in serum of CSU patients and the healthy subjects. *Results.* Serum AGEs and albumin concentrations in CSU patients were significantly lower as compared with the healthy subjects. Serum CRP concentration was significantly higher in patients with CSU than in the controls. Significant positive correlation was observed between AGEs and albumin concentrations in the subjects. *Conclusions.* CSU is not associated with increased circulating AGEs concentrations, despite the enhanced systemic inflammatory response. Paradoxical decrease of serum AGEs concentrations is probably a reflection of lower concentration of “negative acute phase proteins” such as albumin.

## 1. Introduction

Chronic spontaneous urticaria (CSU) is an inflammatory disease, probably caused by an interactive combination of immune, genetic, and environmental factors, including infections [1, 2]. Various changes in levels of immune-inflammatory, coagulation/fibrinolytic, hormonal, and metabolic markers have been reported in CSU patients [3–9].

It has been demonstrated that advanced glycation end-products (AGEs) may be involved in different processes associated with urticarial inflammation, including complement activation, release of inflammatory cytokines, increase of vascular permeability, and procoagulant effects [10–14].

AGEs are formed by nonenzymatic glycation processes and accumulate slowly in tissues and circulation during ageing and more rapidly in diabetes and renal failure [10, 12, 15]. In addition, accelerated increase of AGEs accumulation may be determined in an inflammatory milieu. The accumulating evidence points to AGEs involved in progression of inflammatory and immune-mediated diseases [10, 13]. Interestingly, AGEs upregulate C-reactive protein (CRP) synthesis by human hepatocytes through stimulation of IL-6 [16]. CRP is the marker of systemic CSU activity, reflecting the systemic effects of inflammatory mediators associated with the disease, including IL-6 [5, 17]. Therefore, it seems interesting to test whether chronic inflammation manifested by activated acute phase response may lead to increased AGEs accumulation in CSU patients. Circulating concentrations of AGEs as well as their association with albumin “negative acute phase protein,” CRP “positive acute phase protein” have been investigated in CSU patients and in the healthy subjects.

## 2. Materials and Methods

37 patients with active CSU (26 women and 11 men; mean age: 39 ± 9.5 years) with a median disease duration of 2.9 years were enrolled in the study.

In all cases, any known causes of CSU were ruled in (out) by appropriate investigations. Each patient underwent the following tests: routine laboratory tests (full blood count, urine analysis, ESR, C-reactive protein, serum glucose, hepatic functions, and creatinine), stool (for parasites), hepatitis serology, antinuclear and antithyroid microsomal antibodies, thyroid function tests, chest X-ray, and abdominal ultrasonography. Additionally, dental and ENT consultations as well as the autologous serum skin test (ASST) [1] were performed.

The urticaria activity score (UAS) was estimated during four days and on the blood sampling day and graded as follows: mild (0–8), moderate (9–16), and severe (17–24). The study comprised 20 patients with mild and 17 patients with moderate-severe urticaria symptoms.

None of the examined subjects had taken any oral corticosteroids within 5 weeks or antihistamines within at least 5 days before the study.

The control group consisted of 24 healthy individuals (13 women and 11 men), of comparable age (41.3 ± 8.2 years) and BMI (<30).

The Ethics Committee of the Medical University of Silesia approved the study and written, informed consent was obtained from all the subjects participating.

### 2.1. Blood Collection

Blood samples were taken on fasting, from elbow veins. Sera obtained by centrifugation were stored at −85°C until the tests were performed.

### 2.2. Assay of AGEs

AGEs in the tested sera were determined by flow spectrofluorimetry, according to the method described by Zilin et al. [23]. Conditions were adapted for high-pressure liquid chromatography HPLC-Ultimate 3000 (Dionex, USA) with fluorescent detector RF 2000 (Dionex, USA). The coefficients of variance for intra-assay and interassay were 1.4% and 7.2%, respectively. The method's sensitivity was 0.112 mg/L.

### 2.3. Assay of CRP and Albumin

Serum C-reactive protein (CRP) and albumin concentrations were assayed using Roche/Hitachi cobas c system. Normal lab ranges were lower than 5.0 mg/L and 35–52 g/L, respectively.

### 2.4. Autologous Serum Skin Test (ASST)

Intradermal ASST was performed following the method by Sabroe et al. [1]. Serum-induced red wheal response of diameter greater by at least 1.5 mm than that of a control wheal induced by physiological saline was accepted as positive. Skin prick test with histamine served as a positive control.

### 2.5. Statistical Analysis

The obtained results were presented using basic parameters of descriptive statistics, such as median value, quartile range, mean value, and standard deviation. Normal distribution of data was measured using Shapiro-Wilk's test. Independent data between the groups of patients with CSU and the control group and between CSU patients with mild and moderate-severe symptoms were compared using nonparametric* U* Mann-Whitney test. Spearman's rank test was used for correlations. The *P* < 0.05 was considered statistically significant. Calculations were performed with STATISTICA for Windows 10.0 software (StatSoft, Cracow, Poland).

## 3. Results

### 3.1. Serum AGEs Concentration

**Figure 1:**
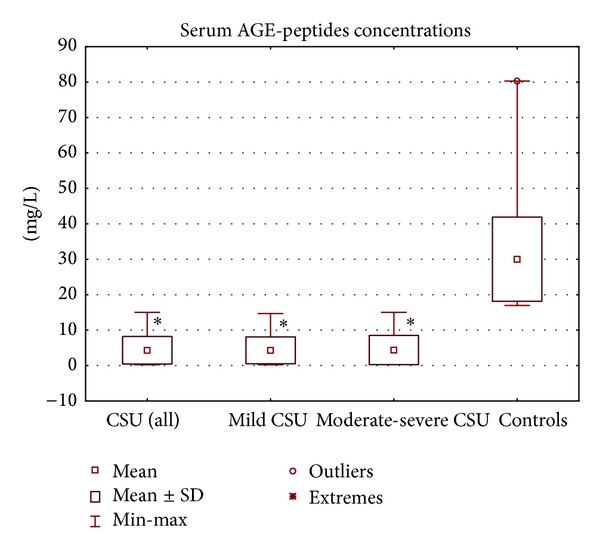
Serum AGEs concentrations in chronic spontaneous urticaria (CSU) patients with different disease activity and in the healthy controls. ∗*P* < 0.05 for comparison: CSU patients versus controls.

Serum concentration of AGEs was significantly lower in CSU patients (as a whole) as compared with the healthy subjects (median: 3.42 versus 26.92 mg/L, *P* < 0.00001; Figure 1). In addition, there were significant differences in serum AGEs concentration between CSU patients with mild and moderate-severe symptoms as compared with the healthy controls (median: 3.54 and 3.25 versus 26.92 mg/L, *P* < 0.00001; Figure 1). Concentration of AGEs in mild CSU group did not differ significantly as compared with severe CSU group (*P* > 0.05; Figure 1). No significant differences in AGEs concentrations between ASST(+) and ASST(−) CSU patients (selected according to the similar UAS) were observed.

### 3.2. Serum Albumin Concentration

**Figure 2:**
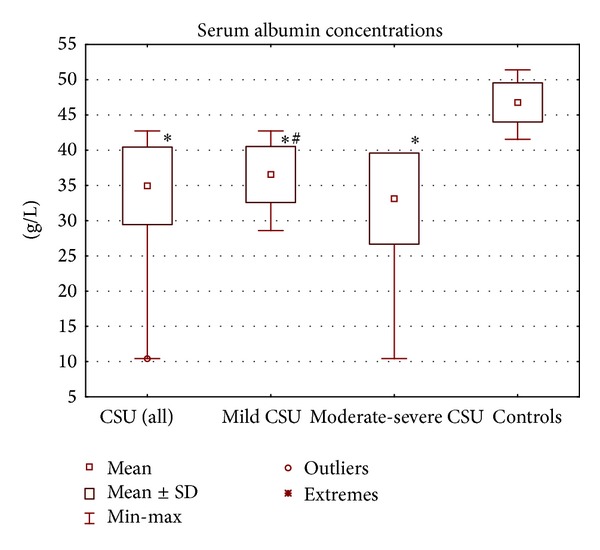
Serum albumin concentrations in chronic spontaneous urticaria (CSU) patients with different disease activity and in the healthy controls. ∗*P* < 0.05 for comparison: CSU patients versus controls. ^**#**^ *P* < 0.05 for comparison: mild CSU versus moderate-severe CSU.

Serum concentration of albumin was significantly lower in CSU patients (as a whole) as compared with the healthy subjects (median: 35.66 versus 47.03 g/L, *P* < 0.00001; Figure 2). There were significant differences in serum albumin concentration between CSU patients with mild and moderate-severe symptoms as compared with the healthy controls (median: 36.66 and 35.31 versus 47.03 g/L, *P* < 0.00001; Figure 2). Concentration of albumin in mild CSU group was significantly higher than in moderate-severe CSU group (median: 36.66 versus 35.31 g/L, *P* = 0.045; Figure 2).

### 3.3. Serum CRP Concentration

Serum CRP concentrations were significantly higher in CSU patients as compared with the healthy subjects (median: 2.85 versus 1.02 mg/L, *P* < 0.00001). There was no significant difference in serum CRP concentration between patients with mild CSU and the healthy controls (median: 1.2 versus 1.02 mg/L, *P* = 0.07); however, CRP concentration in moderate-severe CSU group was significantly higher than in the control group (median: 10.30 versus 1.02 mg/L, *P* < 0.00001). There was also significant difference in serum CRP concentration between patients with mild and moderate-severe CSU (median: 1.2 versus 10.30 mg/L, *P* < 0.00001).

### 3.4. Associations

There was significant negative correlation between albumin and CRP concentrations in CSU patients (*R* = −0.38, *P* = 0.02), but not in the healthy subjects (*R* = −0.05, *P* = 0.79). In addition, significant positive correlation was noted between concentrations of AGEs and albumin (*R* = 0.74, *P* = 0.000000) in the subjects. There were no significant correlations between AGEs and CRP concentrations either in CSU patients (*R* = −0.06, *P* = 0.7) or in the healthy controls (*R* = −0.07, *P* = 0.7).

## 4. Discussion

We present the first evidence to prove that, in CSU patients, regardless of the disease activity/severity, the sera AGEs concentrations were significantly decreased as compared with the healthy subjects. On the other hand, serum CRP concentration was significantly increased as compared with the healthy subjects. At the first glance, it seems paradoxical that serum AGEs concentrations in CSU patients were not increased despite low-grade systemic inflammatory response.

There are some mechanisms, which may account for lower circulating AGEs concentrations in CSU. Firstly, the decreased serum AGEs concentrations can simply be explained by the decline of serum albumin, an important binding site for AGEs [11]. In CSU patients we observed lower serum albumin concentration as compared with the healthy subjects. The contrasting concentration of albumin between CSU patients and the healthy subjects may be explained by activation of acute phase response in CSU.

It is known that, in acute phase response, the synthesis of “positive acute phase proteins,” such as CRP, is increased, whilst the circulating concentrations of the so-called “negative acute phase proteins,” such as albumin, are decreased [17, 18]. Interestingly, it has been demonstrated that IL-6 is able to reduce albumin mRNA expressions [19]. On the other hand, it is known that CSU is associated with increased circulating concentration of IL-6 [5].

In our study, serum CRP concentration was significantly higher in CSU patients as compared with the controls, confirming previous observations regarding acute and chronic urticaria [8, 20, 21]. In addition, significant negative correlation was observed between CRP and albumin concentrations in CSU patients, but not in the healthy subjects.

Interestingly, the positive relationship between albumin and AGEs in the subjects suggests that the decrease in albumin may contribute to lowered AGEs concentration, as most plasma AGEs are irreversibly bound to serum albumin [18]. Therefore, decreased hepatic synthesis of albumins in response to acute phase response or their redistribution secondary to altered vascular permeability may contribute to lower serum albumin concentration resulting in decreased circulating AGEs in CSU patients.

Secondly, it might be interesting to hypothesize that increased vascular permeability may partially contribute to lower serum AGEs concentrations. AGEs accumulation* in vivo* is found throughout the body, including the skin [15]. Further studies are needed to clarify whether lower serum AGEs concentrations are associated with increased accumulation of AGEs at the site of urticarial inflammation.

Thirdly, food intake represents an important source of circulating AGEs [22]. It seems unlikely that diet contributes to lower serum AGEs concentration in CSU patients, because there have not been significant differences in the dietary AGEs intake between the two groups.

Moreover, CSU patients had no documented diseases or complications, which are recognized as associated with changes in AGEs accumulation in the circulation, including diabetes, renal insufficiency, and hepatic failure [10, 15, 23, 24]. On the other hand, formation of AGEs may be accelerated in immunological and respiratory disorders, including allergic rhinitis [25].

In our study, decreased circulating AGEs were not correlated with severity of CSU; serum AGEs concentrations, which were decreased even in mild CSU, were similar to those of moderate-severe CSU.

## 5. Limitation

Despite the small number of subjects enrolled, the present findings have clearly demonstrated a significant decrease of serum AGEs concentration in CSU patients. AGEs in tissue may better reflect the chronic accumulation of AGEs than measuring AGE from serum or plasma. Unfortunately, indices of tissue AGEs accumulation were not measured in our study. It is possible that serum concentration of AGEs is an inadequate reflection of those accumulated in the skin of CSU. Our observation does not exclude the possibility of local AGEs formation/accumulation in the skin, particularly in severe CSU.

## 6. Conclusion

CSU is not characterized by increased circulating AGEs concentrations, despite the enhanced systemic inflammatory response associated with acute phase response.

With regard to the assumed dependency of circulating AGEs concentrations on serum albumin concentration, it should be noted that serum albumin concentration was significantly lower in CSU group as compared to the healthy controls. Correspondingly, a direct, positive relation between AGEs and albumin concentrations was observed. Thus, paradoxical decrease of serum AGEs concentrations is likely to reflect lower concentration of “negative acute phase proteins,” such as albumin.
